# Appropriateness and diagnostic yield of open access gastroscopy in two tertiary centers in South-western Nigeria

**DOI:** 10.4314/ahs.v23i2.44

**Published:** 2023-06

**Authors:** Emuobor A Odeghe, Opeyemi O Owoseni, Evaristus S Chukwudike, Oluwafunmilayo F Adeniyi, Babatunde E Adigun, Ganiyat K Oyeleke, Aderemi O Oluyemi, Olufunmilayo A Lesi

**Affiliations:** 1 Medicine department, University of Lagos/Lagos University Teaching Hospital, Lagos, Nigeria; 2 Medicine department, Federal Medical Centre, Abeokuta, Nigeria; 3 Medicine department, University of Calabar Teaching Hospital, Calabar, Nigeria; 4 Paediatrics department, University of Lagos/Lagos University Teaching Hospital, Lagos, Nigeria; 5 Remay Consultancy and Medical Services, Lagos, Nigeria

**Keywords:** Appropriateness, endoscopy, Nigeria

## Abstract

**Background:**

There is need for the appropriate use of gastroscopy.

**Objective:**

To determine the appropriateness of upper gastrointestinal endoscopy, and its association with significant endoscopy findings in our environment.

**Methods:**

This was a prospective study of subjects who underwent gastroscopy at two centers in south-western Nigeria between August 2020 and August 2021. Indications were classified as either appropriate or inappropriate according to the ASGE guidelines, gastroscopic findings as either significant or not significant, patients as either elderly (≥ 60 years) or not, inpatients or outpatients, and referrals as either gastroenterologist referral, or not.

**Results:**

There were 227 subjects, 131 (57.7%) females, mean age 45 ± 13.7 years. Fifteen percent were elderly, 65.6% were gastroenterologist referrals, 14.1% were inpatients, while 45.8% had co-morbidities. Endoscopy was appropriately indicated in 81.9%, and significant endoscopy findings were detected in 95.6%. Appropriateness was not associated with significant endoscopy findings. The sensitivity, specificity and AUROC of the ASGE guidelines were 10%, 82%, and 0.46 respectively.

**Conclusion:**

According to our study, most procedures are appropriately indicated. However, appropriateness did not determine endoscopy yield. Larger studies are needed to determine the utility of the ASGE guidelines in our environment.

## Introduction

The global increase in availability of open-access endoscopy^1^ has led to a rise in demand for endoscopic procedures. Thus, the American Society for Gastrointestinal Endoscopy (ASGE) has published guidelines on the appropriate use of endoscopy in the evaluation of Gastrointestinal (GI) disease^2^. The purpose of these guidelines is to help endoscopists perform procedures only, when necessary, as the procedures are not without risks.

Data from published works show that up to 14-43% of upper gastrointestinal endoscopy (UGIE) procedures are performed for indications not listed as appropriate by the ASGE^1^, ^3^-^7^ Studies also indicate that the appropriateness of the indication may or may not be dependent on the specialty of the referring physician^6^, ^8^ and that UGIE performed on hospitalized patients were significantly more likely to be appropriate than those carried out on out-patients^8^. There are conflicting data on whether yield is associated with appropriateness of indication, with some studies showing an association^3^,^4^,^9^ and others showing none^7^, ^8^.

The sensitivity and specificity of the ASGE guidelines for abnormal endoscopic findings are 73-89%, and 20-35%^4^,^8^,^10^ respectively, suggesting that the ASGE guidelines are useful in screening patients' indications to determine those who will likely benefit from UGIE.

There has been a marked increase in the number of gastroscopies performed in Nigeria in recent years^11^,^12^, with endoscopic yield ranging between 67-92%^13^-^15^. There is a paucity of local data documenting appropriateness of the procedure, as well as its association with yield in the country. The aims of this study, therefore, were to determine the frequency with which UGIE are performed for an appropriate indication based on the ASGE guidelines, and to determine the association of appropriateness of the indication with the endoscopic yield.

## Patients and methods

This prospective study was carried out in the endoscopy suites of two tertiary care hospitals in the southwestern part of Nigeria from August 2020 to August 2021. Ethical approval was obtained from the Institutional Review Boards of both hospitals before commencement of the study. The study population consisted of all patients aged 16 years and above who were referred for upper GI endoscopy at the endoscopy suites of the two hospitals, and who met the inclusion criteria. Inclusion criteria were referral for UGIE, informed consent given, first-time procedures, and complete procedures. Exclusion criteria were age younger than 16 years, non-consenting, repeat procedures and incomplete procedures. All consecutively presenting, eligible and consenting patients were recruited. Based on a 91.8% prevalence of abnormal findings on UGIE in Lagos^15^, the minimum sample size was determined to be 116.

Before the commencement of each procedure, a questionnaire specifically designed for this study was administered to each participant. Data collected were socio-demographics, the indication for the procedure, gastrointestinal symptoms present, treatment with proton pump inhibitor, the specialty of the referring physician, domicile of patient, presence of co-morbidity, use of potentially gastro-toxic drugs (e.g., aspirin and non-steroidal anti-inflammatory drugs (NSAIDs), results of *Helicobacter pylori* (*H. pylori*) testing using the rapid urease test, and endoscopic findings.

Age was categorized as ≥ 60 years or not; indication for the procedure was classified as either appropriate or not according to the ASGE guidelines; the specialty of the referring physician was classified as either gastroenterologist (medical), or not; and the domicile of patient was classified as either outpatient or inpatient. The co-morbidities sought included hypertension, diabetes mellitus, cirrhosis, ischemic heart disease (IHD), chronic kidney disease (CKD), stroke, asthma, hepatitis B and hepatitis C infections (HBV, HCV), and human immunodeficiency virus infection (HIV). The outcome variable was the presence of significant endoscopy findings (SEF), which was defined as any of the following: erosions (oesophageal, gastric, duodenal), gastroduodenal ulcers, upper GI masses and polyps, varices, strictures and foreign bodies. All other findings were categorized as non-significant. Some of the indications that were classified as appropriate were persistent dyspepsia despite an appropriate trial of therapy, upper abdominal symptoms associated with other symptoms/signs suggesting structural disease, or new-onset symptoms in patients > 50 years, upper gastrointestinal bleeding, and gastro-oesophageal reflux disease symptoms that persist or recur despite appropriate therapy^2^.

Statistical analysis was performed using R version 4.0.2 software (The R Foundation for Statistical Computing). Continuous data, such as age, were expressed as mean while categorical data, such as gender, and significant endoscopic findings, were expressed as percentages. The diagnostic yield was calculated as the ratio of significant endoscopy findings to the number of procedures performed. The frequency at which gastroscopy was performed for appropriate and inappropriate indications was calculated. The association of appropriateness of indication and other categorical variables with significant endoscopy finding was determined and expressed as odds ratios with 95% confidence intervals, using univariate and multivariate logistic regression. The sensitivity and specificity of the ASGE guidelines for the detection of significant findings on gastroscopy were also calculated. A p-value of <0.05 was considered to be significant.

## Results

### Demographics of study participants

Two hundred and twenty-seven subjects were recruited into the study, comprising 131 females (57.7%), and 96 males (42.3%) (female/male ratio 1.4:1), with mean age 45 ± 13.7 years. Thirty-four (15%) were aged 60 years and older, 149 (65.6%) were referred by a gastroenterologist, 32 (14.1%) were inpatients, and 104 (45.8%) had co-morbidities which were hypertension (80, 35.2%), diabetes (24, 10.6%), cirrhosis (13, 5.7%), HBV infection (4, 1.8%), asthma (3, 1.3%), and others (CKD, HIV, IHD, HCV, sickle cell anaemia (SCA), systemic lupus erythematosus (SLE), tuberculosis (TB), Parkinson's disease, neurofibromatosis; 1 each (0.4%)).

Endoscopy was appropriately indicated in 186 (81.9%). Appropriateness of indication was associated with inpatient status and older age, but not with mean age, gender, gastroenterologist referral or presence of comorbidities. The appropriate indications were persistent dyspepsia despite an appropriate trial of therapy (71, 31.3 %), upper abdominal symptoms associated with other symptoms/ signs suggesting structural disease, or new-onset symptoms in patients > 50 years (63, 27.8%), upper gastrointestinal bleeding (UGIB) (35, 15.4%), gastro-oesophageal reflux disease (GERD) symptoms that persist despite appropriate therapy (11, 4.8%), chronic diarrhoea for suspected small bowel disease (3, 1.3%), diagnosis/ treatment of varices in portal hypertension (2, 0.9%) and progressive dysphagia/odynophagia (1, 0.4%), while the only inappropriate indication was upper abdominal pain/ discomfort that had not received a trial of therapy (41, 18.1%). These are shown in table 1.

**Table 1:** General characteristics of the study participants and association with appropriateness

Characteristic	All (%)	Appropriate (%)	Inappropriate (%)	p-value	cOR (95% CI)
Age, mean (SD)	45.0 (13.7)	45.6 (14.1)	42.5 (11.3)	0.1	1.02 (0.991.04)
Male	96 (42.3)	74 (39.8)	22 (53.7)	0.2	0.6 (0.3-1.1)
Age ≥ 60	34 (15)	32 (17.2)	2 (4.9)	0.05	4.1 (1.2-25.7)
Gastroenterology referral	149 (65.6)	124 (66.7)	25 (61)	0.6	1.3 (0.6-2.6)
Inpatient	32 (14.1)	32 (17.2)	0 (0)	0.002	N/A
NSAID	55 (24.2)	45 (24.2)	10 (24.4)	1.0	0.99 (0.5-2.3)
H.pylori positivity	133 (58.6)	111 (59.7)	22 (53.7)	0.5	1.3 (0.6-2.5)
Co-morbidity	104 (45.8)	89 (47.9)	15 (36.6)	0.3	1.6 (0.8-3.3)
Significant endoscopy findings	217 (95.6)	177 (95.2)	40 (97.6)	0.5	0.5 (0.03-2.7)

Data is presented as mean ± standard deviation or n (%); cOR = crude Odds ratio, NA= not applicable

The diagnostic yield was calculated as the ratio of SEF to the number of procedures. Significant endoscopic findings were present in 217 subjects, giving an overall diagnostic yield of 95.6%. The yields for appropriate and inappropriate indications were 95.2% (177/186) and 97.6% (40/41) respectively; appropriateness of indication was not significantly associated with significant endoscopy findings. There was no significant difference in the mean age of the subjects with or without SEF (45.3 ± 13.8 versus 39.8 ± 11.4, p=0.2). The presence of significant endoscopy findings was not associated with gender, Gastroenterologist's referral, hospitalization, co-morbidity or any of the indications, as shown in table 2.

**Table 2:** Association of significant endoscopy findings with subject demographics and symptoms

Characteristic	SEF+	SEF-	cOR (95%CI)	aOR (95%CI)
ASGE appropriate indications	177 (81.6)	9 (90)	0.5 (0.03-2.7)	0.7 (0.03-10.7)
Male	93 (42.9)	3 (30)	1.8 (0.5-8.3)	1.2 (0.3-7.3)
Age ≥ 60 years	33 (15.2)	1 (10)	1.6 (0.3-30.3)	1.8 (0.2-38.0)
Gastroenterology referral	143 (65.9)	6 (60)	1.3 (0.3-4.7)	1.8 (0.4-7.7)
Inpatient	30 (13.8)	2 (20)	0.6 (0.2-4.4)	0.3 (0.04-2.8)
Co-morbidity	101 (46.5)	3 (30)	2.0 (0.6-9.6)	1.9 (0.4-10.2)
Gastro-toxic drug	53 (24.4)	2 (20)	1.3 (0.3-8.7)	1.1 (0.2-8.2)
Currently smoking	12 (5.5)	0 (0)	NA	NA
Any alcohol intake	48 (22.1)	1 (10)	2.6 (0.5-47.7)	2.1 (0.3-43.9)
Herbal drug intake	110 (50.7)	3 (30)	2.4 (0.7-11.4)	2.5 (0.6-14.1)
H. pylori positivity	126 (58.1)	7 (70)	0.6 (0.1-2.2)	0.6 (0.1-2.2)
Persistent dyspepsia despite appropriate therapy	68 (31.3)	3 (30)	1.06 (0.3-5.1)	0.9 (0.1-7.1)
Upper gastrointestinal bleeding	35 (16.3)	0 (0)	NA	
Dyspepsia with structural disease, or new-onset symptoms in patients > 50 years	59 (27.2)	4 (40)	0.6 (0.2-2.3)	0.4 (0.1-2.8)
Persistent GERD symptoms	11 (5.1)	0 (0)	NA	
Progressive dysphagia or odynophagia	0 (0)	1 (10)	NA	NA
Diagnosis/treatment of varices in portal hypertension	2 (0.9)	0 (0)	NA	
Chronic diarrhoea	2 (0.9)	1 (10)	0.08 (0.01-1.9)	NA

Data is presented as n (%); cOR, aOR = crude and adjusted Odds ratios; NA= not applicable

The significant endoscopy findings were gastric/duodenal erosions (179, 78.9%), oesophageal erosions (67, 29.5%), gastroduodenal ulcers (42, 18.5%), oesophageal varices (14, 6.2%), upper GI masses (11, 4.8%) and upper GI polyps (4, 1.8%). All the oesophageal varices and upper GI masses were found in the appropriately indicated procedures. Endoscopy was normal in 10 (4.4%). These findings were not mutually exclusive.

The sensitivity, specificity and AUROC of the ASGE indications for the detection of significant endoscopic findings were 10%, 81.6%, and 0.46 respectively.

## Discussion

In this study of the appropriateness of gastroscopy in a sample of Nigerian patients, we determined that 82% of procedures were appropriate, while 18% were inappropriate. Similar rates were documented by Keren et al^1^ (84%), Mudawi et al^3^ (86%), Rajan et al^4^ (85.9%), and Chan and Goh16 (88.3%) in studies from Israel, Sudan, India and Malaysia respectively. However, lower rates of appropriateness were reported by Tachi et al^6^ (58.9%), Aljebreen et al 8 (69%) and Gupta et al^17^ (24.6%). Our high rate of appropriateness may be explained by our centres being tertiary level hospitals, where only the most distressed patients with significant symptomatology are referred, and thus appropriate referrals are likely to be made irrespective of specialty of the physician in charge of the patients' care. It may also be due to the majority of referrals being from gastroenterologists because endoscopists reportedly have the highest rates of appropriately referred patients compared to others 3,16.

We found that hospitalized patients, and older patients had significantly more appropriate referrals than outpatients and younger patients, as were also documented by Mudawi et al^3^, Aljebreen et al^8^, and Gupta et al 17. Our results may be a reflection of a greater disease burden in hospitalized patients and the older population. We did not find appropriateness to be associated with the specialty of the referring physician, as the rates of appropriateness did not differ between referrals from gastroenterology and non-gastroenterology specialty practitioners. This is similar to the finding by Gupta et al^17^, but is in contrast to the study by Chan and Goh who found appropriateness to be associated with endoscopists and primary care providers (PCPs) 16. The reason for our finding may be our method of categorizing referrals as either from gastroenterologists or non-gastroenterologists, thus we may have included referrals from other endoscopists such as surgeons as non-gastroenterologist. There was no association of appropriateness with gender as in other studies 3, 17, 18

The most frequent appropriate indications were persistent dyspepsia despite an appropriate trial of therapy, dyspepsia associated with features suggestive of structural disease, or new-onset symptoms in patients > 50 years, and upper GI bleeding, while the only inappropriate indication was dyspepsia in patients who had not received a trial of therapy, similar to findings reported in other studies^4^, ^8^ ^16^. This is hardly surprising as dyspepsia and UGIB are among the most frequent indications for gastroscopy in Nigeria^14^, ^19^, ^20^.

The overall diagnostic yield in our study was 95.6% similar to findings in other local studies^15^, ^21^. Lower yields however were demonstrated in other studies 6, 17. In our study, yield did not differ between appropriate and inappropriate procedures, as was also found by Azzam et al^18^. In contrast, some studies reported significantly higher yields when procedures were performed for appropriate indications^3^, ^4^, ^17^. Erosions, which were our most frequent finding, were present in equal proportion in both appropriately and inappropriately indicated procedures. This may be explained by the frequent use of NSAID/ gastro-toxic drugs, and herbal drugs as well as the high rates of *H. pylori* infection, as these are factors that are well known to cause erosions. Our findings are similar to those documented from other local and regional studies^14^,^21^,^22^.

On univariate and multivariate logistic regression analyses, we did not find SEF to be associated with male gender, older age, hospitalization or gastroenterology referral. While some studies found SEF to be associated with male gender, GI referral, inpatient status, and older age, others did not find such association^4^, ^6^ ^8^ ^17^, ^18^.

Our study demonstrates that the ASGE indications have a low sensitivity and moderate specificity for significant endoscopic findings, in contrast to the studies by Aljebreen et al, and Rajan et al^4^, ^8^. The reason for the low sensitivity may be the lack of significant association of yield with appropriateness, as well as the similar endoscopy findings in both appropriately and inappropriately indicated procedures. This may suggest that they may not be useful for prioritising gastroscopy indications in our environment. Notwithstanding that all the oesophageal varices and gastroduodenal masses were found in the appropriately indicated procedures, other significant findings were present in the inappropriate procedures and these may have been missed if the guidelines were strictly adhered to, and a more liberal use of endoscopy has been advocated^1^.

Limitations include that it was a public hospital-based study which was carried out in only the south-western part of the country.

In conclusion, less than a fifth of gastroscopy procedures were inappropriate based on ASGE indications in two open access endoscopy systems in southwestern Nigeria. The appropriateness of indications did not predict the presence of significant findings on endoscopy. Larger studies are needed to determine the utility of the ASGE guidelines in our resource-limited setting.

## Figures and Tables

**Table 3:** Endoscopy findings of the study participants and association with appropriateness

Characteristic	All (%)	Appropriate (%)	Inappropriate (%)	p-value
Gastric/duodenal erosions	179 (78.9)	144 (77.4)	35 (85.4)	0.3
Oesophageal erosions	67 (29.5)	54 (29)	13 (31.7)	0.7
Gastroduodenal ulcers	42 (18.5)	35 (18.8)	7 (17.1)	0.8
Oesophageal varices	14 (6.2)	14 (7.5)	0 (0)	0.99
Upper gastrointestinal mass	11 (4.8)	11 (5.9)	0 (0)	0.99

Data is presented as n (%)

**Figure 1:**
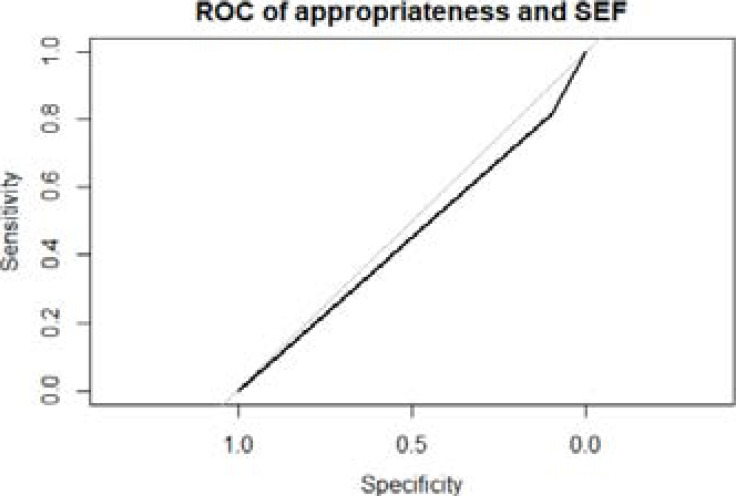
Receiver operating characteristic curve of appropriateness and significant endoscopy findings
